# Clinical evaluation of non-contact infrared thermometers

**DOI:** 10.1038/s41598-021-99300-1

**Published:** 2021-11-11

**Authors:** Stacey J. L. Sullivan, Jean E. Rinaldi, Prasanna Hariharan, Jon P. Casamento, Seungchul Baek, Nathanael Seay, Oleg Vesnovsky, L. D. Timmie Topoleski

**Affiliations:** 1 Division of Applied Mechanics, Office of Science and Engineering Laboratories, Center for Devices and Radiological Health, United States Food and Drug Administration, 10903 New Hampshire Ave, Silver Spring, MD 20993 USA; 2 University of Maryland Baltimore County, Baltimore, MD USA

**Keywords:** Fever, Biomedical engineering, Mechanical engineering

## Abstract

Non-contact infrared thermometers (NCITs) are being widely used during the COVID-19 pandemic as a temperature-measurement tool for screening and isolating patients in healthcare settings, travelers at ports of entry, and the general public. To understand the accuracy of NCITs, a clinical study was conducted with 1113 adult subjects using six different commercially available NCIT models. A total of 60 NCITs were tested with 10 units for each model. The NCIT-measured temperature was compared with the oral temperature obtained using a reference oral thermometer. The mean difference between the reference thermometer and NCIT measurement (clinical bias) was different for each NCIT model. The clinical bias ranged from just under − 0.9 °C (under-reporting) to just over 0.2 °C (over-reporting). The individual differences ranged from − 3 to + 2 °C in extreme cases, with the majority of the differences between − 2 and + 1 °C. Depending upon the NCIT model, 48% to 88% of the individual temperature measurements were outside the labeled accuracy stated by the manufacturers. The sensitivity of the NCIT models for detecting subject’s temperature above 38 °C ranged from 0 to 0.69. Overall, our results indicate that some NCIT devices may not be consistently accurate enough to determine if subject’s temperature exceeds a specific threshold of 38 °C. Model-to-model variability and individual model accuracy in the displayed temperature were found to be outside of acceptable limits. Accuracy and credibility of the NCITs should be thoroughly evaluated before using them as an effective screening tool.

## Introduction

Non-contact Infrared Thermometers (NCITs) are being used as a temperature measurement tool for screening and isolating potentially infected people with elevated temperature in healthcare settings, ports of entry (PoEs), and in other settings during the Coronavirus Disease 2019 (COVID-19) pandemic^1^. Elevated temperature greater than or equal to 38 °C (42 CFR 70.1) is one of the symptoms exhibited by persons with COVID-19. To successfully screen and track people with elevated temperature, it is essential that accurate temperature measurements are made, and that the thermometer outputs are correctly interpreted.

ASTM E1965-98(2016)^2^ and ISO 80601-2-56(2017)^3^ are both FDA-recognized voluntary consensus standards used by device manufacturers to evaluate the performance of NCITs by i) testing the accuracy of the device against a standard blackbody source (BBS) and ii) performing a clinical study to evaluate the accuracy and effectiveness of the device in clinical settings. NCITs are FDA class-II medical devices (21 CFR 880.2910) approved under product code FLL^4^. FDA’s 510(k) premarket notification database shows that more than 20 NCITs have been cleared by the FDA in the past 3 years.

NCITs do not measure the core body temperature directly but are designed to correlate with a reference body site temperature, such as the oral temperature^2,3^. The forehead skin surface temperature is measured based upon detection of infrared radiant energy from the surface of the skin. The temperature of the forehead skin surface is lower than reference body site temperature. Therefore, manufacturers typically use a proprietary algorithm and hardware design features to compensate for the difference between the forehead skin surface temperature and the reference body site temperature—the “adjusted mode,” typically referred to as “subject mode” for most NCITs. The algorithm used to adjust the temperature also may compensate for other factors such as variations in room temperature, skin emissivity, and clinical and hardware biases.

NCITs are generally not as accurate as contact thermometers.

The accuracy of the temperature measured by NCITs can be affected by the following factors:

1. Inaccuracy of the sensor measuring the forehead skin surface temperature (Δ_sensor_).
2. Inaccuracy in the algorithm which is used to predict the reference body site temperature from forehead skin surface temperature (Δ_algorithm_).
3. Inaccuracy in the forehead skin surface temperature caused by use errors such as incorrect distance and angle between the NCIT and the forehead skin surface (Δ_user_).
4. Inaccuracy in the forehead skin surface temperature due to cooling or heating of the forehead skin surface by external factors such as sweating, exposure to sun, and wind currents (Δ_environmental_).

NCIT standards (ASTM E1965 and ISO 80601-2-56) state that the laboratory accuracy using a black body source (BBS) shall be within ± 0.3 °C. These standards do not include a specific requirement for clinical accuracy. Although NCITs may be the primary tools for temperature screening during a pandemic, clinical studies have reported mixed performance in terms of their accuracies^5–8^. Several studies evaluated the performance of NCITs in a pediatric population. Hayward et al.^7^ measured the mean difference between NCIT and axillary thermometer temperatures to be − 0.14 °C with a 95% confidence interval of − 0.21 to − 0.06 °C. Franconi et al.^6^ performed a comparative observational study on a pediatric population and observed a significantly higher mean difference of − 0.41 °C. Khan et al.^8^ quantified the mean difference between NCIT and temporal artery thermometer in adults to be ± 0.26 °C. Conversely, Dante et al.^5^ observed that the mean difference between axillary and forehead temperatures (− 0.06 °C) was not statistically significant. These studies focused primarily on a pediatric population, which represents only a subset of the general population, thus limiting the applicability of the results to the general population that will be subjected to mass screening during a pandemic.

The Canadian Agency for Drugs and Technologies in Health performed a review of clinical studies to understand the clinical effectiveness of NCIT devices^9^. Their analysis found the mean temperature difference between NCITs and reference thermometers varied between − 0.1 °C and 0.66 °C. While some studies expressed conclusions in favor of the utilization of infrared skin thermometry, others stated that NCIT accuracy is unsatisfactory. Chen et al.^10^ performed a prospective observational study during the novel coronavirus outbreak of 2019 to compare the accuracy and precision of forehead temperature with tympanic temperature. The mean difference ranged from − 1.72 to − 0.56 °C. Bitar et al.^11^ in a similar clinical study reported the sensitivity of NCITs to vary widely from approximately 4–90%. Overall, previous studies using clinical data from hospitals and transit centers have been inconclusive regarding the use and effectiveness of NCITs as a screening method during SARS and influenza outbreaks. These contradictory findings may be attributed to limitations such as small subject sample size, insufficient credibility in the reference thermometer, and the use of a limited number of NCIT brands and models.

During prior disease outbreaks and pandemic events, the Centers for Disease Control and Prevention (CDC) recommended the use of NCITs as a screening tool at PoEs^12^. The continued use of NCITs in a screening capacity presents the need to ensure that the temperature measurement accuracy claims made by the manufacturers are valid and that the NCIT measurements are able to effectively identify people with elevated temperatures at or above the CDC threshold^1^.

The objective of this study was to evaluate, analyze, and report the accuracy of various commonly-available NCIT models in a large-scale controlled clinical study comprised of both afebrile and febrile adult subjects. Oral temperatures from more than 1000 subjects were obtained using one clinical-grade oral reference contact thermometer and compared with six different models of NCITs. The difference between the NCIT and reference thermometer was analyzed. Based on the results of the clinical study, the adequacy of NCITs to detect the actual oral temperature is presented.

## Methods

A total of 1113 human subjects were enrolled in the study at the University Health Center located at the University of Maryland, College Park. This clinical study was approved by the FDA Institutional Review Board (IRB). All experiments were performed in accordance with relevant guidelines and regulations. Before conducting the tests, informed consent was obtained from all participants.

### Non-contact infrared thermometers

Six different commercially available NCIT models from different manufacturers that measure temperature at the center of the forehead were selected (Table 1). All the selected NCIT models provided the option to choose oral temperature as the reference site temperature. Ten units of each model were purchased from commercial vendors. NCITs were divided into 10 identical sets; each measurement set contained one unit of each of the six different NCIT models, labeled A through F. The accuracy of these models stated in the manufacturers’ instructions for use ranged from ± 0.2 °C to ± 0.3 °C. Thermometers were cleaned and prepared according to the manufacturer’s instructions for use and had fresh batteries installed prior to testing.

**Table 1:** Manufacturer’s specifications from instructions for use.

Unit	A	B	C	D	E	F
Human/object	Both	Both + room	Both	Human	Both	Both + room
Ambient operating temp limits (°C)	16–40	10–40	10–40	15–40	0–50	16–40
Body temp measurement limits (°C)	34–43	32–42.9	34.4–42.2	34.4–42.2	32–42.5	34–42.5
Body temp displayed	Oral	Oral	Oral	0.5 °C below rectal equivalent to oral	“Body” equivalent to oral	Oral
% Humidity operating limits	0–85; non condensing	< 85	0–85	15–95	10–90	15–93
Accuracy (°C)	± 0.2 (for 36–39C); ± 0.3 (outside meas. Range)	± 0.2	± 0.2 (for 36–39C); ± 0.3 (for 33–35.9C); ± 0.3 (for 39.1–42.2C)	± 0.2 (inside meas. Range); ± 0.3 (outside meas. Range)	± 0.3	± 0.2 (for 36–39C) ± 0.3 (for 34–35.9C, 39.1–42.5C)

### Study population

The characteristics of the subjects are as follows. 60% of the subjects were female and the rest were male. In terms of age, 49% of the subjects were between 18 and 20 years, 44% between 21 and 30 years, and the rest above 30 years. In terms of ethnicity, 47% were white, 14% were African–American, 27% were Asian, 7% were Hispanic, and other ethnicities constituted 5%.

### Subject inclusion

Any subject above the age of 18 who could sit for at least 15 min with permitted breaks as needed and follow the study instructions was included.

### Subject exclusion

Any subject under the age of 18 or persons not willing or able to remain seated during temperature measurements was excluded.

### Experimental protocol

The usage protocol for the individual NCIT models was designed according to the instructions for use. The same Welch Allyn oral thermometer (SureTemp Plus 690, Welch Allyn, San Diego, CA) with a measurement accuracy of ± 0.1 °C in monitor mode was used to measure the oral temperature (i.e., sublingual pocket temperature) of each subject. All NCIT measurements used the oral reference body site setting.

A common user error is taking a measurement at the incorrect distance from the target. The manufacturer-recommended distance between the forehead and the NCIT varied among the models and ranged from 0.5 inches to 6 inches. One model, F, incorporated a distance assurance mechanism into its design. To ensure that the proper measurement distance specified by the manufacturer was consistently maintained for NCITs A-E, each was fitted with a custom positioning adapter. Single-use cotton swabs of specific lengths as recommended by the manufacturers for each thermometer model were then inserted into the adapter to produce a controlled fixed distance between the device and the target. The cotton swabs were positioned to not interfere with the temperature readings.

Room temperature and humidity were recorded (Kestrel 4500 NV, Weather Republic LLC, Downingtown, PA) for each subject session. Data for this study were collected over an 18 month period. Room temperature was monitored during the entire study duration and ranged from 20.2 to 29.3 °C. During the typical single subject’s measurement time of 15 min, the room temperature variation did not exceed 1 °C. If the room temperature and humidity fell outside the manufacturer’s operating range, the data was taken but not included in the analysis. More details about the exclusions are provided later. If the subject’s forehead was visibly moist (perspiration), the NCIT measurement area was dried by blotting gently with a paper towel. For each measurement, the NCIT infrared (IR) detector was positioned at the same location on the center of the subject’s forehead. The subjects were in the indoor study environment for at least 20 min before the measurements began. The total duration of measurement for each subject was not more than ~ 15 min. The time gap between first and second trial was ~ 10 min.

The same operator made temperature measurements for a specific subject. For a specific subject, we used the same set of infrared thermometers for all the trials. The sequence of temperature measurements was as follows:

1. Temperature was measured by placing the oral reference thermometer under the subject’s tongue, in monitor mode, for 3 min as specified by the instructions for use.
2. Temperature measurements were made using all 6 models of NCITs starting from Model A and ending with Model F (Trial #1). Measurements were obtained immediately following the reference temperature.
3. A second oral reference thermometer measurement (step #1) and second set of NCIT measurements (step #2) were made on the same subject (Trial #2). NCIT measurements were obtained immediately following the reference temperature.

### Statistical plan and data analysis

The following analyses were performed on the NCIT temperature data for trial #1 and trial #2 independently:

1. Differences between the NCIT temperature (*T*_*NCIT*_) and reference thermometer (*T*_*ref*_) were determined,
  1 $$\Delta T\left( {n,i,m} \right) = T_{NCIT} \left( {n,i,m} \right) - T_{ref} \left( {n,i,m} \right).$$
  where *n* is the trial number (*n*=1,2); *i* is the NCIT model (*i*=A:F), and *m* is the individual subject (m=1:M). M is the total number of subjects.
2. The Clinical Bias (as per ASTM E1965 and ISO 80601-2-56 standards), i.e., the average difference for a NCIT model, was calculated,
  2 $$\left( {\Delta T_{average} } \right)_{i,n} = \left( {\sum \, \Delta T\left( m \right)} \right)_{i,n} /M$$
3. Analysis of variance (ANOVA) combined with post-hoc pairwise analysis (Tukey’s Test) was performed using R statistical programming language (free software, R Foundation for Statistical Computing) to evaluate whether the temperature measurements obtained from the reference thermometer and each NCIT model were statistically different from one another.
4. A correlation analysis was performed to evaluate the strength of the relationship between ΔT and T_ref_. This analysis was performed to determine the relationship between the accuracy of the NCITs and the oral temperature.
5. Sensitivity and specificity estimates were made for a range of threshold temperatures, *T*_*threshold*_ (37.0, 37.1…38.0 °C).
  3 $$\begin{aligned} Sensitivity\; & = \frac{{\left( {\# \;of\;subjects\;where\;T_{ref} \ge T_{threshold} \;AND\,T_{NCIT} \ge T_{threshold} } \right)}}{{\left( { \, \# \;of\;subjects\;where\;T_{ref} \ge T_{threshold} } \right)}} \\ Specificity\; & = \frac{{\left( {\# \;of\;subjects\;where\;T_{ref} < T_{threshold} \;AND\;T_{NCIT} < T_{threshold} } \right)}}{{\left( {\# \;of\;subject\;where\;T_{ref} < T_{threshold} } \right)}} \\ \end{aligned}$$

A cumulative total of 13,356 temperature measurements were made during Trials #1 and #2 using the six different NCIT models. For these temperature measurements, exclusions were made using the following prioritized criteria. Once data was excluded it was not reevaluated for exclusion by a subsequent exclusion criteria.

1. Temperature was measured not following the clinical protocol (18 subjects)
2. T_ref_ not recorded during Trial #1 or Trial #2 (33 subjects)
3. T_ref_ < 36.1 °C (40 subjects)
4. Ambient humidity was less than the operating relative humidity stated by the manufacturer (136 subjects; Only data for those models not meeting the manufacturer’s ambient relative humidity were excluded).
5. NCIT temperature not recorded (4 subjects; All other recorded data retained)

## Results

The T_ref_ measurements for 1022 subjects ranged from 36.1 to 40.3 °C. Number of subjects with T_ref_ between 36.1 and 37.2 °C was 866 and 854 for Trial #1 and #2, respectively. Number of subjects with T_ref_ between 37.2 and 38.0 °C was 107 and 109 for Trial #1 and #2, respectively. Number of subjects with a T_ref_ ≥ 38 °C was 49 and 59 for Trial #1 and #2, respectively. The average temperature was the same (36.9 °C) for Trial #1 and Trial #2.

**Figure 1:**
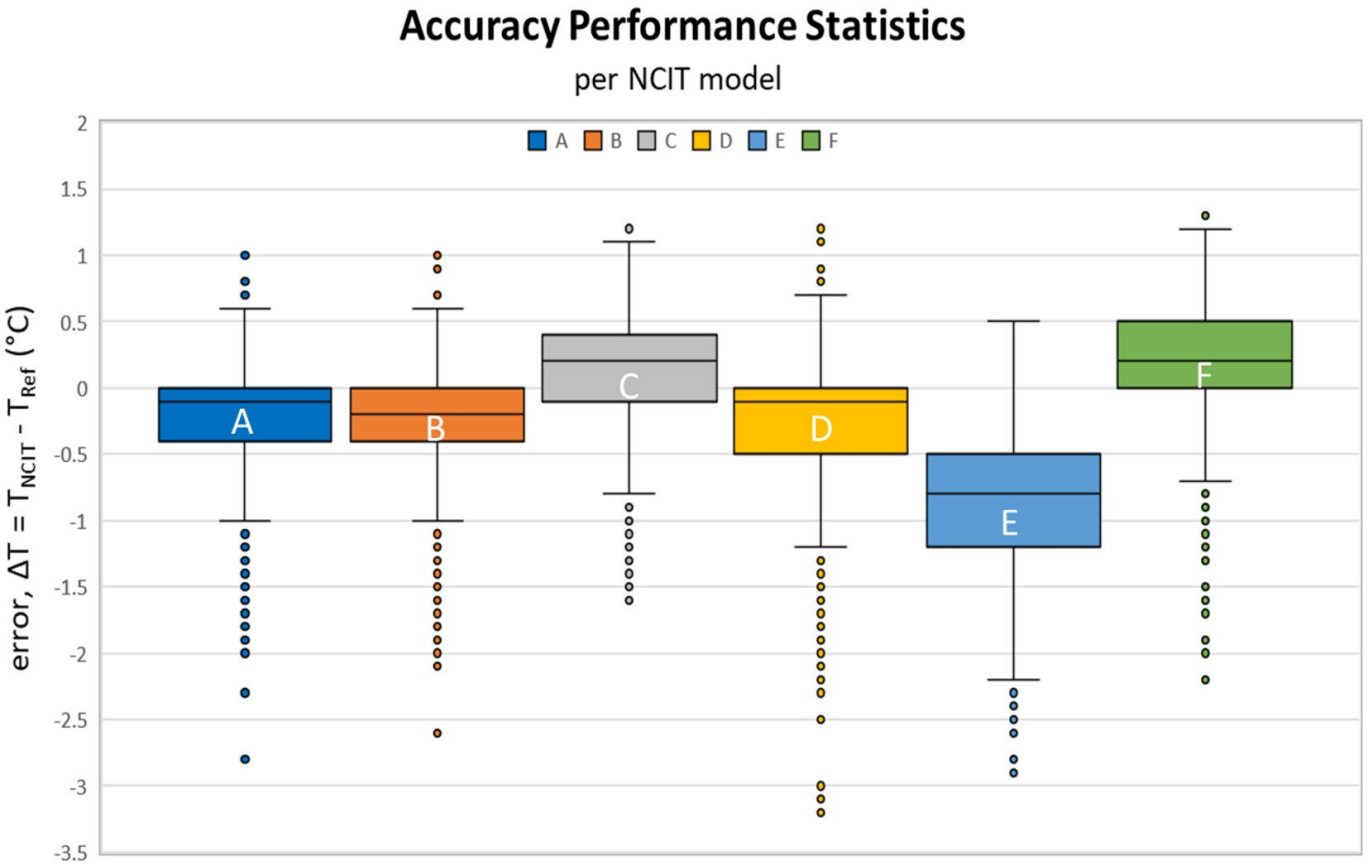
Accuracy performance statistics for each NCIT model for Trial #1. The midline indicates the median, the box top captures 25% of the data above the median and the box bottom captures 25% of the data below the median. The whiskers (error bars) represent that maximum and the minimum ΔT. The circles represent outlier data.

Clinical bias was calculated for all six NCIT models (Table 2). The clinical bias (Trial #1) ranged from under-reporting the temperature by − 0.87 °C to over-reporting the temperature by 0.21 °C. Model E had the largest clinical bias (− 0.89 °C) while Model C had the smallest clinical bias (0.14 °C). All six NCIT models had relatively large standard deviations compared to mean (Table 2 and Fig. 1). The 5th percentile value for ΔT was between − 1.9 and − 0.5 °C.

**Table 2:** ΔT (°C) for all six NCIT models.

ΔT = T_NCIT_—T_Ref_ (°C)												
NCIT models	A		B		C		D		E		F	
Trial	#1	#2	#1	#2	#1	#2	#1	#2	#1	#2	#1	#2
Clinical bias (average)	− 0.23	− 0.28	− 0.22	− 0.24	0.15	0.14	− 0.32	− 0.31	− 0.87	− 0.89	0.21	0.23
Standard deviation	0.46	0.45	0.43	0.40	0.42	0.39	0.61	0.54	0.53	0.54	0.48	0.43
95th percentile	0.40	0.30	0.40	0.30	0.70	0.70	0.50	0.40	− 0.10	− 0.20	0.90	0.80
5th percentile	− 1.10	− 1.20	− 1.00	− 1.00	− 0.70	− 0.60	− 1.50	− 1.30	− 1.80	− 1.90	− 0.60	− 0.50

**Figure 2:**
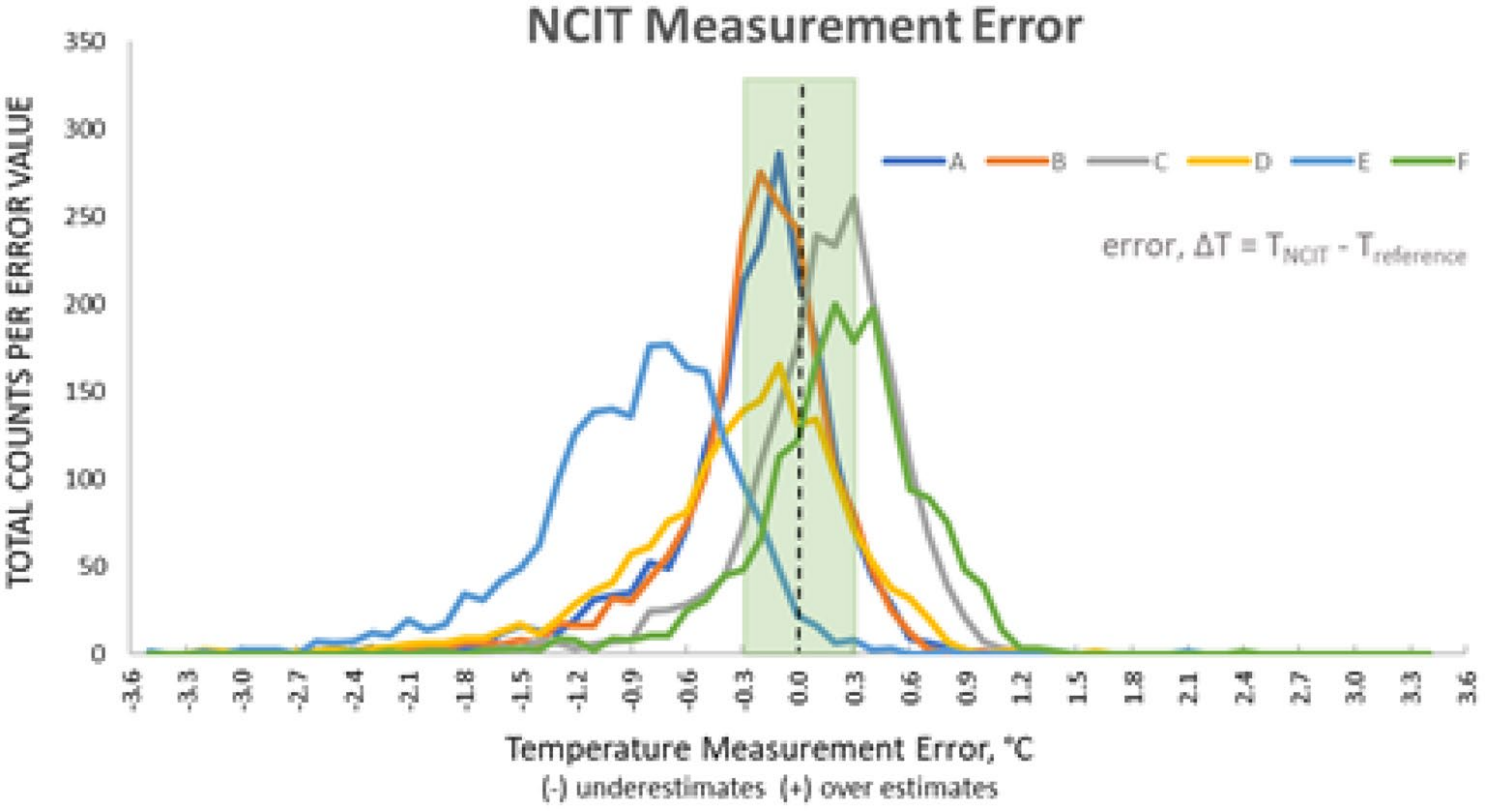
Total counts per error value, per NCIT model for Trial #1 and #2. Green area indicates ± 0.3 °C laboratory accuracy zone; dashed black line indicates the zero error line.

For the six NCIT models, the mode value for ΔT varied between − 0.7 and 0.4 °C (Fig. 2). For all models, more than 48% of the clinical measurements fell outside of the manufacturer’s accuracy claim (Table 3).

**Table 3:** Number of measurements outside of the manufacturer’s accuracy claim.

NCIT Models	A		B		C		D		E		F	
Stated accuracy for measurement range	± 0.2 °C (36 °C to 39 °C) ± 0.3 °C (< 36 °C; > 39 °C)		± 0.2 °C		± 0.2 °C (36 °C to 39 °C) ± 0.3 °C (< 36 °C; > 39 °C)		± 0.2 °C		± 0.3 °C		± 0.2 °C (36 °C to 39 °C) ± 0.3 °C (< 36 °C; > 39 °C)	
Trial	#1	#2	#1	#2	#1	#2	#1	#2	#1	#2	#1	#2
Total number of readings	1021	1022	1022	1022	1022	1022	884	884	1019	1019	886	886
Number of ΔTs outside of the stated accuracy	493	523	503	497	606	538	564	527	874	891	557	545
% of ΔT outside of the stated accuracy	48.3	51.2	49.2	48.6	59.3	52.6	63.8	59.6	85.8	87.4	62.9	61.5

The difference between reference temperature and NCIT temperature was statistically significantly different for all six models.

Overall, temperatures measured by each NCIT model were found to be statistically significantly different from one another (Table 4). Model pairs A and B for Trial #1, and model pairs A and D for Trial #2, were the only instances where pairs were not found to be significantly different.

**Table 4:** ANOVA comparisons between NCIT models for the difference between the reference and the NCIT. “O” stands for not being statistically different between two NCIT models, while “X” stands for being statistically different.

Comparison	Trial #1	*p* value, (Trial 1)	Trial #2	*p* value, (Trial 2)
A and B	O	0.384	X	0.035
A and C	X	< 0.001	X	< 0.001
A and D	X	< 0.003	O	0.592
A and E	X	< 0.001	X	< 0.001
A and F	X	< 0.001	X	< 0.001
B and C	X	< 0.001	X	< 0.001
B and D	X	< 0.001	X	0.016
B and E	X	< 0.001	X	< 0.001
B and F	X	< 0.001	X	< 0.001
C and D	X	< 0.001	X	< 0.001
C and E	X	< 0.001	X	< 0.001
C and F	X	< 0.004	X	< 0.001
D and E	X	< 0.001	X	< 0.001
D and F	X	< 0.001	X	< 0.001
E and F	X	< 0.001	X	< 0.001

Intra-model variability in ΔT measurement among the ten different NCIT units of the same model are presented in Table 5. Analysis showed that only models C and F reported intra-model consistency. For the other models, the intra-model variability in ΔT was large and the temperature measurements were inconsistent. Statistical significance is *p* < 0.05.

**Table 5:** ANOVA results for consistency in ΔT between ten NCIT units of the same model for each trial independently. “O” stands for no statistical difference between ten NCIT units, while “X” indicates statistical difference between ten NCIT units.

Model	Trial #1	*p* value, (Trial 1)	Trial #2	*p* value, (Trial 2)
A	X	< 0.001	X	< 0.001
B	X	0.024	X	0.044
C	O	0.144	O	0.090
D	X	< 0.001	X	< 0.001
E	X	< 0.001	X	< 0.001
F	O	0.394	O	0.406

**Figure 3:**
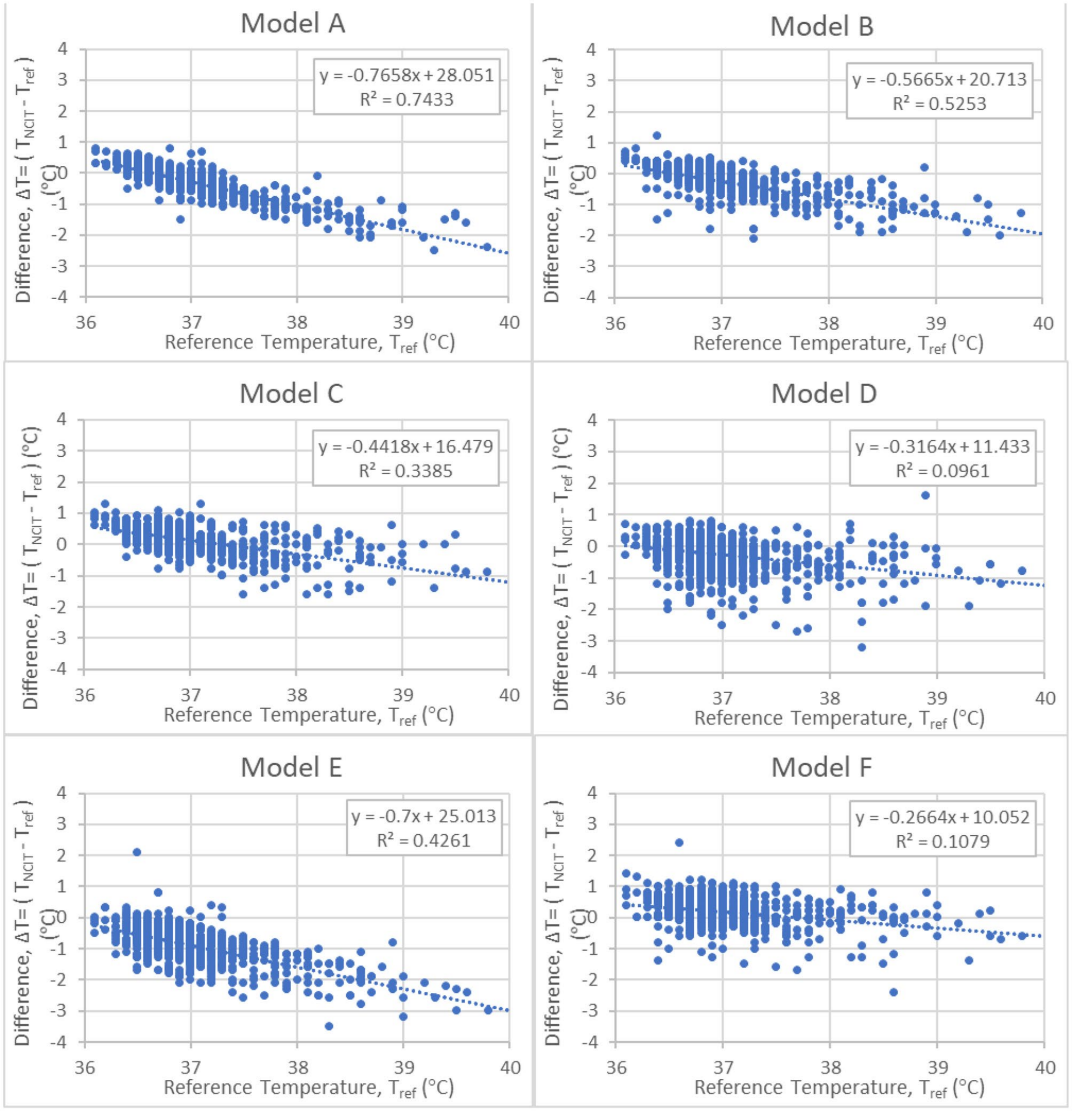
ΔT (°C) as a function of T_ref_ (°C).

The correlation between ΔT and T_ref_ showed that the difference, ΔT, changed as a function of T_ref_ for all NCIT models (Fig. 3). The slope for the linear regression varied between 0.35 and 1.1. Statistical significance is *p* < 0.05.

**Figure 4:**
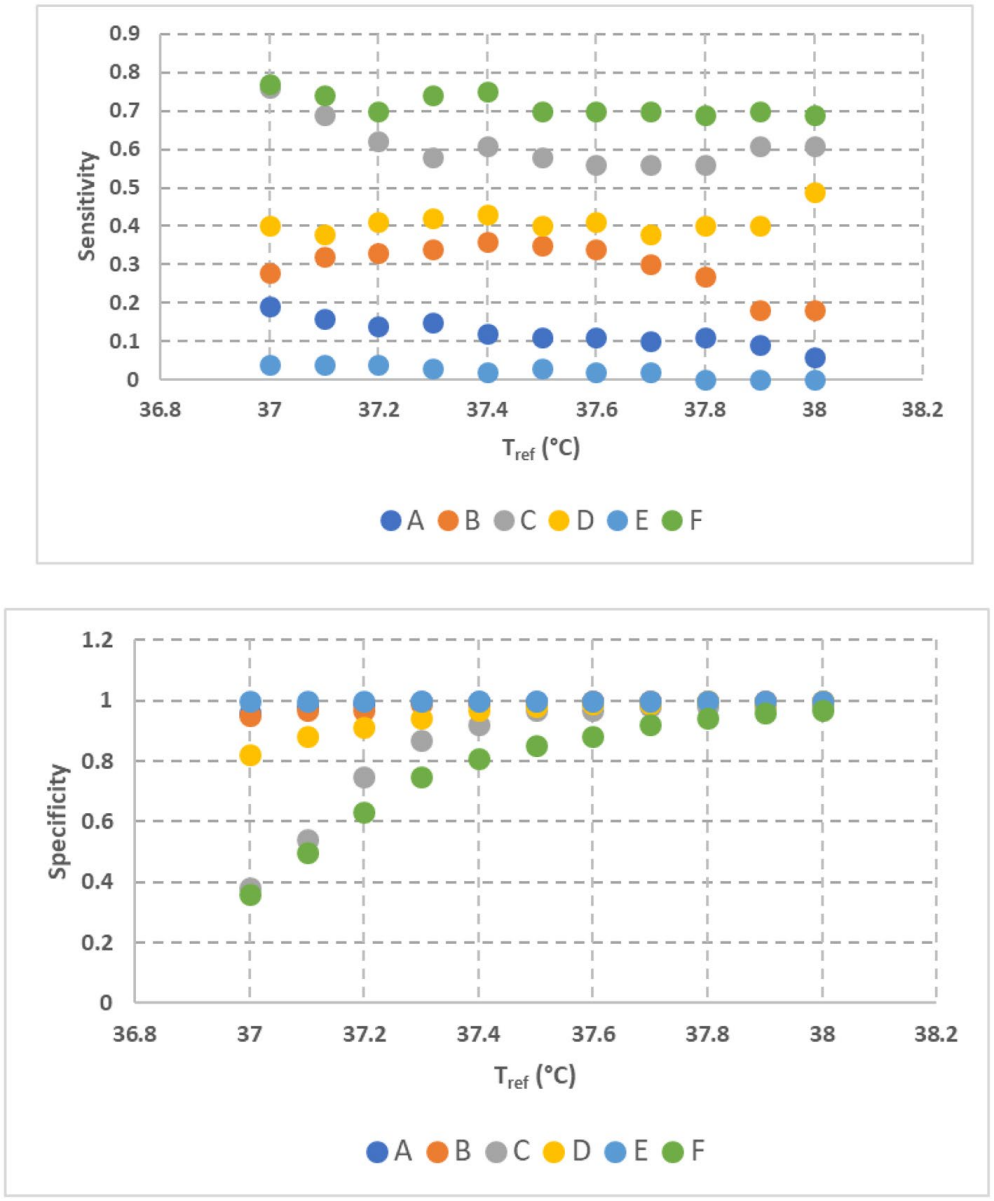
Sensitivity and specificity of all NCIT models obtained from Trial #1 dataset

Sensitivity was dependent on the threshold temperature (Fig. 4). As the threshold temperature increased, the sensitivity decreased. Specifically, the sensitivity of the NCIT models for measuring 38 °C ranged from 0 (model E) to 0.69 (Model F).

Figure 4 shows that specificity of the NCIT models for measuring 38 °C ranged from 0.97 (model F) to 1 (Model A,B,D,E). Specificity was also dependent on the measured temperature. As the subject’s temperature increased, the specificity increased by a considerable manner (Fig. 4).

## Discussion

The clinical performance of commercially available NCITs was assessed using 1022 adult subjects in a controlled setting. The accuracy of the NCITs in a clinical setting was evaluated using:

1. The clinical bias and the temperature measurement inconsistency represented as standard deviation (Table 2).
2. The differences in the temperature measurements between the NCIT and reference thermometer (Fig. 1).
3. Number of measurements falling outside of the accuracy stated by the manufacturer (Table 3).
4. Sensitivity and specificity for predicting a subject’s temperature above 38 °C (Fig. 4).

This study incorporated a very large sample size (> 1000 subjects) and used multiple NCIT models. Our results indicated that both clinical bias and uncertainty for the six NCIT models exceeded the stated accuracy in their product labeling. Only one of the six NCIT models (Model C) had a clinical bias within the manufacturer’s stated accuracy (Table 3). Depending upon the NCIT model, 48–88% of the individual temperature measurements were outside of the labeled accuracy stated by the manufacturers (Table 3). Even for Model C, which had the lowest clinical bias, 50% of the individual measurements fell outside the stated accuracy. Model E, with the highest clinical bias, had 88% of the data falling outside the stated accuracy. Statistical analysis also showed that the NCIT measurements from all six models were different from the corresponding reference thermometer measurements. Overall, all our metrics highlight challenges with measuring a subject’s temperature and resulting credibility issues with NCIT measurements in a controlled setting according to the manufacturer’s instructions for use.

The accuracy of NCIT devices are currently evaluated using the ASTM E1965 and ISO 80601-2-56 standards. Both standards require the laboratory error to be within ± 0.3 °C. Laboratory error measures the temperature against a standardized BBS under controlled conditions and does not include errors introduced by the proprietary software algorithm, user error, physiological variability, and environmental factors. Therefore, in a clinical setting, the variability in the NCIT temperature measurement is expected to be greater than the laboratory error. Our study illustrated that the error (ΔT) can range from − 3 to + 2 °C in extreme cases, with the majority of the errors ranging from − 2 to + 1 °C (Figs. 2, 3) outside of the manufacturer’s stated accuracy (Table 3). Our study protocol was designed to minimize the inaccuracies due to user error (Δ_user_) and environmental factors (Δ_environmental_). In a real-world setting (e.g., transit centers, PoEs, pre-clinical triage, and other screening locations), the additional inaccuracies and variabilities will only increase the error in NCIT-measured body temperature unless the measurement protocols control for these factors.

Our results showed that the error in the NCIT readings appears to depend upon the subject’s temperature (Fig. 3). The linear regression of the NCIT measurement error with respect to the subject’s oral temperature for all NCIT models showed a negative slope. As the subjects’ temperatures increase, the NCIT readings transition from overestimating to underestimating the oral temperature.

There are several potential explanations for the negative slope. One possibility is that the reference thermometer was inaccurate. Another possibility is that the offset algorithms used to convert forehead temperature measured by NCIT to oral temperature were inaccurate. Our reference thermometer was calibrated for accuracy across the operating temperatures (Attachment A). Our calibration data showed that the accuracy of the reference thermometer was not dependent on measured temperature. In addition, the reference temperature was obtained using a contact probe (oral) which tends to be more reliable compared to non-contact measurement. Therefore, our data indicate that the root-cause for this negative slope can likely be attributed to the offset algorithm in the NCITs. Further analysis should be done understand and address the limitations of the existing offset algorithms in the NCITs.

Based on the sensitivity analysis (Fig. 4), our study showed that some of the NCITs are likely to generate significant false negative readings when used for fever detection. The sensitivity of the NCIT models at 38 °C, the CDC defined temperature threshold^1^, ranged between 0 and 0.69. Four of the six NCIT models had sensitivity less than 0.5 with two of them below 0.1. Therefore, four of the six models had a false negative rate of more than 50%. Because of the high probability for producing false negative readings close to the CDC threshold, these NCITs are an unreliable stand-alone temperature screening tool.

Our study included over one thousand subjects and six different NCIT models (ten units of each model for a total of sixty thermometers). The measurements were obtained under well-controlled conditions; however, we recognize that the study has several limitations. Subjects under the age of 18 were not included. The number of subjects with temperature measurements ≥ 38 °C was approximately 5% of the total sample. Nonetheless, the statistical analysis showed there were sufficient subjects to analyze the adequacy of the NCIT accuracy. While there are many commercially available NCITs, for practical purposes, we focused our study on six NCIT models from different manufacturers over a wide price range. We chose these NCITs because they all targeted the center of the forehead. While we evaluated the inter- and intra-model variability in accuracy, other confounding clinical factors such as sex, age, skin tone, and weight were not considered and should be evaluated in subsequent studies.

While oral temperature measurement is widely used in public settings as a surrogate for core temperature, it may not provide a robust measure for core temperature like the pulmonary artery (PA) temperature. The purpose of this study was not to correlate oral to PA temperatures, but to evaluate the ability of the NCITs to report temperatures correlating to oral temperatures, as advertised in their literature, instructions for use, and as an operational mode in all the NCITs tested. No NCIT (tested in this study) had a true core temperature mode. While PA temperature measurement would be ideal, similar comparisons have been made between infrared cameras and oral thermometry^13^.

Overall, our results indicate that some NCIT devices may not be consistently accurate enough to be used as a stand-alone temperature measurement tool to determine if the temperature exceeds a specific threshold (e.g., 38 °C) in an adult population. Model-to-model variability and individual model accuracy in the displayed temperature are a major source of concern. Users should be aware of the consequences of false negatives and false positives when using NCITs as a screening tool.

In addition, it is critical to follow the manufacturer’s instructions for use to minimize inaccuracies due to user error and other environmental factors in order to ensure the optimal results from these devices. The FDA published a fact sheet that contains recommendations to be followed to minimize some of the inaccuracies in the NCIT measurements^14^. Factors affecting NCIT temperature measurement and their interpretations should be considered when developing the temperature measurement protocol and screening criteria.

## Supplementary Information

Supplementary Information.

## Abbreviations

BBS: Black body source
NCIT: Non contact infrared thermometer
IRB: Institutional Review Board
FDA: Food and Drug Administration
CDC: Center for Disease Control and Prevention
CDRH: Center for Devices and Radiological Health
PoE: Port of Entry
ANOVA: Analysis of variance
