# Supplementary material for: Clinical evaluation of non-contact infrared thermometers
Source: Sci Rep. 2021 Nov 11;11:22079. doi: 10.1038/s41598-021-99300-1 (PMC8586154; doi:10.1038/s41598-021-99300-1)
Supplement: Supplementary file 1 — Supplementary Information. [file 41598_2021_99300_MOESM1_ESM.docx]

**Attachment A**

The accuracy of the reference oral thermometer used in the clinical study was evaluated using a water-bath. A water-bath was set to a range of temperature from 30 °C to 40 °C. The water-bath was then measured using the oral reference thermometer used in the clinical study and a calibrated reference thermistor probe. The thermistor bead temperature sensor was designed in collaboration with Alpha Technics (Irvine, CA). The probe works as a part of a commercially available temperature measurement system (T-View system, Alpha Technics, Irvine, CA). It has a nominal resolution of 0.001 °C.

Overall, the temperature measured by the clinical reference thermometer matched the thermistor probe within 0.05 °C (Figure A1). The manufacturer stated accuracy limit for the reference thermometer is ± 0.1 °C

**
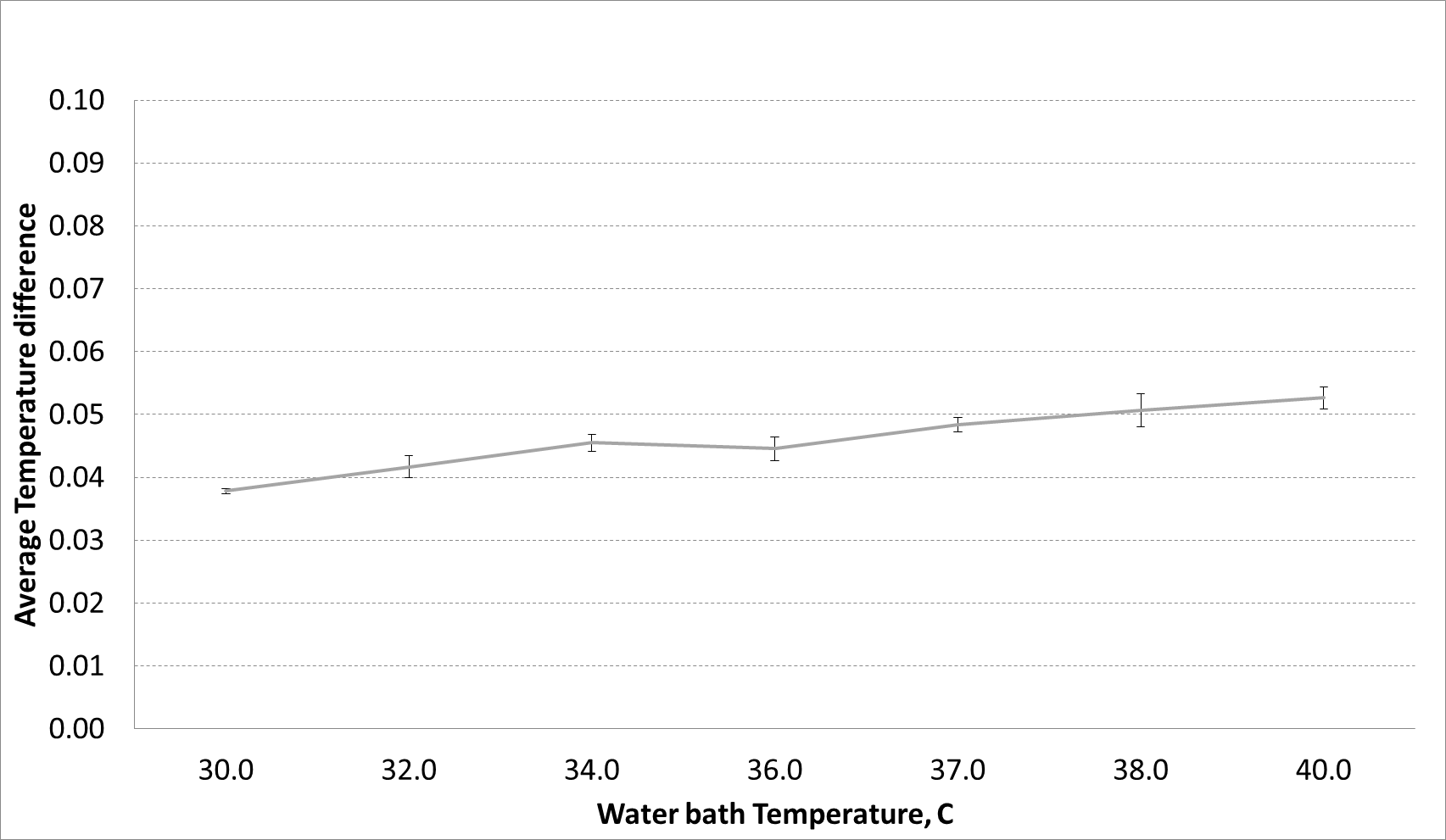
**

Figure A1: Average temperature difference between the Welch Allyn oral thermometer (i.e., T_ref_ for the clinical study) and the thermistor probe as a function of water-bath temperature.
